# Planes, Trains, and Automobiles: Use of Carbon Dioxide Monitoring to Assess Ventilation During Travel

**DOI:** 10.20411/pai.v7i1.495

**Published:** 2022-02-25

**Authors:** Jennifer L. Cadnum, Heba Alhmidi, Curtis J. Donskey

**Affiliations:** 1 Research Service, Louis Stokes Cleveland VA Medical Center, Cleveland, Ohio; 2 Geriatric Research, Education, and Clinical Center, Louis Stokes Cleveland VA Medical Center, Cleveland, Ohio; 3 Case Western Reserve University School of Medicine, Cleveland, Ohio

**Keywords:** SARS-CoV-2, COVID-19, ventilation, carbon dioxide, travel, restaurant

## Abstract

**Background:**

Travel poses a risk for transmission of severe acute respiratory syndrome coronavirus 2 (SARS-CoV-2) and other respiratory viruses. Poorly ventilated indoor settings pose a particularly high risk for transmission.

**Methods:**

We used carbon dioxide measurements to assess adequacy of ventilation during 5 trips that included air travel. During selected parts of each trip that involved indoor settings, we monitored carbon dioxide levels every 1 minute and recorded peak levels and the number of people present. Carbon dioxide readings above 800 parts per million (ppm) were considered an indicator of suboptimal ventilation.

**Results:**

Carbon dioxide levels remained below 800 ppm during train rides to and from the airport and inside airports except in a crowded boarding area with ~300 people present. Carbon dioxide levels exceeded 800 ppm inside the airplanes, but the air was filtered with high efficiency particulate air filters. Carbon dioxide levels remained below 800 ppm in common areas of a hotel but exceeded 800 ppm in a hotel room with 2 to 3 occupants and in a fitness center with 3 people exercising. In restaurants, carbon dioxide levels increased above 800 ppm during crowded conditions with 24 or more people present and 75% or more seat occupancy.

**Conclusion:**

Our results suggest that ventilation may be sufficient to minimize the risk for airborne transmission in many situations during travel. However, ventilation may be suboptimal in some areas or under certain conditions such as in hotel rooms or when restaurants, fitness centers, or airplane boarding areas are crowded. There is a need for larger scale studies to assess the quality of ventilation in a wide range of community settings.

## INTRODUCTION

Travel poses a substantial risk for transmission of severe acute respiratory syndrome coronavirus 2 (SARS-CoV-2) and other respiratory viruses because people are often required to be in close proximity for prolonged periods [1–4]. Transmission of SARS-CoV-2 has been reported on airplanes, with most secondary cases occurring in passengers sitting within a few rows of the source patient [5]. However, the risk for airborne transmission during a flight may be relatively low in comparison to other enclosed spaces because the cabin air is exchanged every 3 to 4 minutes and the recirculated air is filtered through high-efficiency particulate air (HEPA) filters [5–8]. Other situations that might place travelers at risk include crowded settings inside airports, using public transportation, staying in hotels, and eating in restaurants. Inadequate ventilation in these indoor spaces increases the risk for airborne transmission of respiratory viruses [1–3]. However, limited information is available on the adequacy of ventilation in many areas encountered during travel.

Carbon dioxide levels are commonly used as an indicator of the adequacy of ventilation in occupied indoor environments [1, 2, 4–9, 10]. The concentration of carbon dioxide in outdoor air is approximately 400 parts per million (ppm) versus approximately 40,000 ppm in exhaled breath [9]. Thus, carbon dioxide levels rise in occupied spaces that are poorly ventilated. Relatively little information is available on levels of carbon dioxide in indoor settings associated with travel. Therefore, we measured carbon dioxide levels during 5 trips that included use of public transportation and an overnight hotel stay to assess the adequacy of ventilation during different parts of the journey.

## METHODS

The study was approved as a quality improvement project by the Research and Development Committee and Infection Control Committee of the Louis Stokes Cleveland VA Medical Center. During 5 trips that included air travel, carbon dioxide levels were monitored using an IAQ-MAX CO2 Monitor and Data Logger (CO2Meter, Inc) that monitors carbon dioxide levels, temperature, relative humidity, and barometric pressure with a sampling time of 1.5 seconds. All readings were collected anonymously. Each of the flights were 2 to 3 hours in duration. During selected parts of each trip that involved indoor settings, we recorded carbon dioxide levels with the data logging interval set to record levels every 1 minute and noted the minimal and maximal levels, and the number of people present. Peak carbon dioxide levels were graphed for multiple indoor areas during use of public transportation, during stays in a hotel, and in restaurants.

Public transportation monitoring sites included riding a train to and from the airport, in different locations inside the airport, and in the airplane during boarding, in-flight at cruising altitude, and during deplaning while parked at the gate. Hotel monitoring sites included the lobby, hallways, fitness center, breakfast room, and hotel room with 2 or 3 occupants. Carbon dioxide levels were monitored during 4 indoor restaurant meals.

The Centers for Disease Control and Prevention (CDC) has recommended that carbon dioxide readings above 800 ppm in buildings may be considered an indicator of suboptimal ventilation requiring intervention [11]. Peak levels above 800 ppm were therefore considered an indicator of suboptimal ventilation for the number of occupants present [11, 12].

## RESULTS

**Figure 1:**
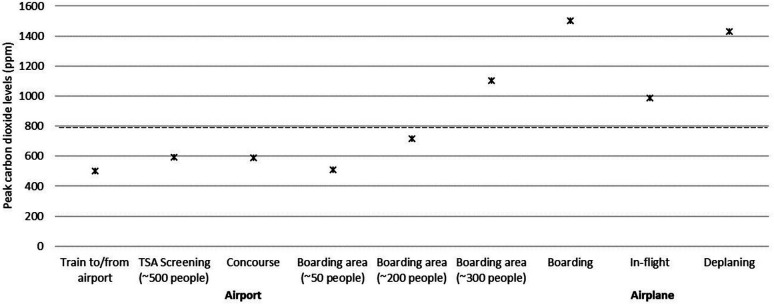
**Air Travel.** Peak carbon dioxide levels in different areas during travel on a train to and from the airport and inside the airport and airplane. Peak levels above 800 PPM (dotted line) were considered an indicator of suboptimal ventilation for the number of occupants present. PPM, parts per million. TSA, Transportation Security Administration.

Figure 1 shows the peak carbon dioxide levels in different areas during travel on a train to and from the airport and inside the airport and airplane. During 1-hour train rides to and from the airport, the carbon dioxide levels ranged from 410 to 515 ppm with between 3 and 20 passengers in a large train car (23 meters long). During 7 ½ total hours of monitoring in 3 airports, the carbon dioxide levels remained under 800 ppm except in a crowded boarding area; the peak carbon dioxide level was 1104 ppm in the boarding area when approximately 300 people were crowded together, including passengers exiting a plane and waiting to board. In airport bathrooms with up to 8 people present, carbon dioxide levels remained under 600 ppm. During the cruising phase of the flights, the average carbon dioxide level was 832 ppm with a peak of 988 ppm. During 4 flights, the air conditioning was on throughout boarding and deplaning and the carbon dioxide levels rose substantially, peaking at 1500 ppm and 1430 ppm, respectively. During 1 flight, the air conditioning system was not turned on until midway through boarding; the carbon dioxide levels peaked at 3405 ppm with the air conditioning off and decreased to ∼1500 when the air conditioning was turned on.

**Figure 2:**
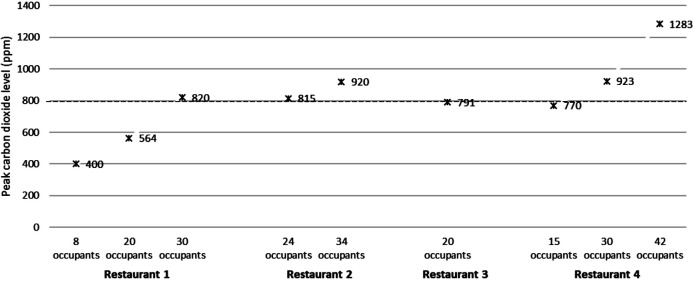
**Restaurants.** Peak carbon dioxide levels in 4 restaurants during periods with different numbers of people present, including restaurant patrons and staff. Peak levels above 800 PPM (dotted line) were considered an indicator of suboptimal ventilation for the number of occupants present. PPM, parts per million.

Figure 2 shows the peak carbon dioxide levels inside 4 restaurants during periods with different numbers of people present, including restaurant patrons and staff. In 3 of the 4 restaurants, the carbon dioxide levels peaked at a concentration above 800 ppm. The carbon dioxide levels in the restaurants increased as the number of people increased. The highest level recorded was 1283 ppm in restaurant 4 when 42 people were present; the peak carbon dioxide level in restaurant 4 was 770 ppm when 15 people were present. When 42 people were present in restaurant 4, approximately 90% of seats in the restaurant were occupied. In restaurants 1 and 2, approximately 75% of seats were occupied when the peak carbon dioxide levels were recorded. All 4 restaurants had incoming and outgoing air vents.

**Figure 3:**
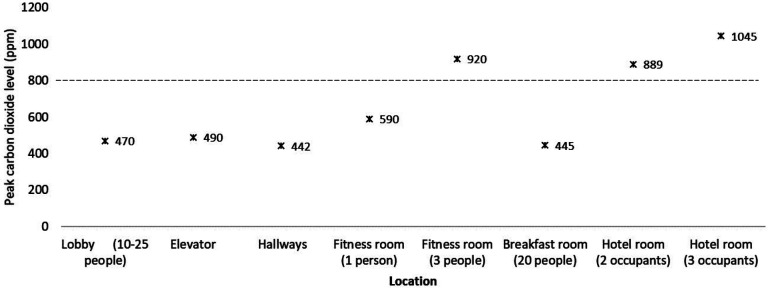
**Hotel.** Peak carbon dioxide levels at various locations inside a hotel. Peak levels above 800 PPM (dotted line) were considered an indicator of suboptimal ventilation for the number of occupants present. PPM, parts per million.

**Figure 4:**
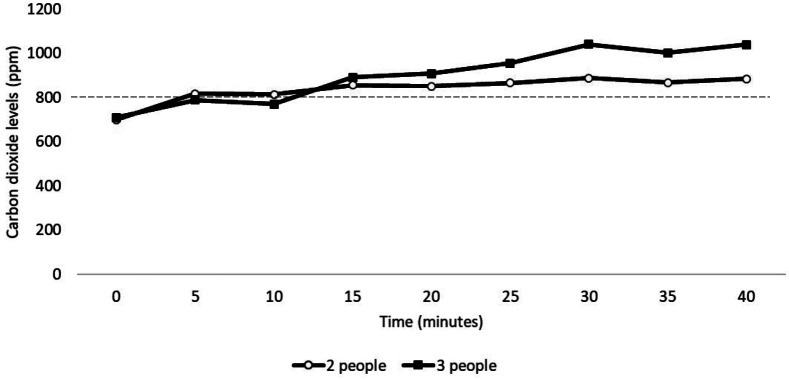
Carbon dioxide levels in a hotel room with 2 or 3 occupants. The occupants entered the room at time 0. Peak levels above 800 PPM (dotted line) were considered an indicator of suboptimal ventilation for the number of occupants present. PPM, parts per million.

Figure 3 shows the peak carbon dioxide levels at various locations inside a hotel. The peak carbon dioxide level was below 800 ppm when 1 person was in the fitness center but rose above 800 ppm when 3 people were exercising. In the hotel room, carbon dioxide levels peaked above 800 ppm when either 2 or 3 people were present. Figure 4 shows the change in carbon dioxide levels for the first 40 minutes after room entry with 2 or 3 occupants. For multiple episodes of monitoring, carbon dioxide levels peaked at approximately 30 minutes after room entry and remained stable while monitoring for up to 8 hours.

## DISCUSSION

Ensuring adequate ventilation is an important measure to reduce the risk for airborne transmission of SARS-CoV-2 and other respiratory viruses [1]. Our findings suggest that ventilation may be sufficient to reduce the risk for airborne transmission in many situations during travel. However, our results also highlight the fact that ventilation may be suboptimal in some areas or under certain conditions such as in hotel rooms or when restaurants, fitness centers, or airplane boarding areas are crowded.

Air travel poses a potential risk for transmission on the airplane, inside the airport, and during use of public transportation to and from the airport. We found that carbon dioxide levels during train rides to and from the airport and while inside the airport remained below 800 ppm except in a very crowded boarding area with ∼300 people present. During the flights, the average carbon dioxide level was above 800 ppm with a peak level of 988 ppm. However, given that re-circulated air is HEPA-filtered, the risk for airborne transmission is generally considered relatively low [5–8]. The substantially higher carbon dioxide levels during boarding and deplaning is consistent with a prior study [13], and suggests that ventilation may be less optimal at these times. It has been recommended that the air conditioning systems of airplanes should be operated during boarding and during disembarkation to minimize risk for SARS-CoV-2 transmission [9]. During 4 flights, the air conditioning system was in operation during boarding and deplaning; during boarding for 1 flight the air conditioning system was not in operation and carbon dioxide levels increased to greater than 3,000 ppm.

Eating in restaurants has been associated with COVID-19 infection in several studies [14–17]. Transmission clusters in restaurants have been linked to both suboptimal ventilation and directed airflow from air conditioners or fans facilitating long-distance dispersal of respiratory droplets [14, 15]. In addition, the need to remove masks while eating increases the risk for transmission. In healthcare facilities, clusters of COVID-19 have also been associated with sharing of meals in breakrooms by healthcare personnel [18]. For 3 of the restaurants studied, carbon dioxide levels were recorded during different periods when the number of room occupants differed substantially. For each of these restaurants, the carbon dioxide levels peaked above 800 ppm when 24 or more people were present and approximately 75% or more of the seats were occupied. For all restaurants, carbon dioxide levels were below 800 ppm when 20 or fewer people were present. These results suggest that ventilation in the studied restaurants was adequate to minimize the risk for airborne transmission except under crowded conditions. Similarly, elevated carbon dioxide levels have been reported with overcrowding of small conference rooms or offices in a hospital setting [4].

Although transmission of SARS-CoV-2 has been reported in hotels [19–21], limited information is available on the potential for transmission in hotels. In the current study, carbon dioxide levels remained below 800 ppm in most common areas. The carbon dioxide levels increased above 800 ppm in a hotel room with 2 to 3 occupants and in a small fitness center when 3 people were exercising. Previous studies have demonstrated transmission of SARS-CoV-2 in gyms [22, 23]. Gyms could pose a relatively high-risk for transmission due to increased respiration and aerosol particle production associated with exercise [22–24]. Blocken *et al* [23] demonstrated that the addition of portable air cleaners to the existing ventilation system of a gym was effective in reducing aerosol particle buildup.

Our study has several limitations. The sample size was small. Carbon dioxide levels were only assessed during 5 trips involving air travel with 2 types of airplanes, 1 type of train, and 1 hotel. However, the carbon dioxide levels inside the airplane were similar to levels measured inside airplanes during a previous study that included multiple flights and types of airplanes [13]. Elevated carbon dioxide levels have not been directly linked to SARS-CoV-2 transmission risk. However, poorly ventilated indoor spaces are generally considered high-risk areas [1]. The train cars were not full of passengers during the trips to and from the airport. Therefore, evaluations are needed in trains and buses during trips with full occupancy. We did not include an assessment of carbon dioxide levels in motor vehicles used to drive to and from the train station in the current study. Information regarding carbon dioxide levels in motor vehicles has been collected and will be reported separately. Finally, our investigation did not address measures other than ventilation that might affect transmission risk during travel (eg, compliance with facemasks and physical distancing).

## CONCLUSION

Monitoring of carbon dioxide levels provides a useful indicator of the adequacy of ventilation in occupied indoor environments. Our results suggest that ventilation during travel may be adequate to minimize the risk of airborne transmission in many settings. However, our findings also suggest that ventilation may be suboptimal in indoor areas such as hotel rooms with 2 to 3 occupants and in crowded restaurants, fitness centers, and airplane boarding areas. Avoiding areas or situations with suboptimal ventilation or developing interventions to improve ventilation or filter air may reduce the risk for viral transmission in these settings. Given the relatively small scale of our study, there is a need for larger studies to assess the quality of ventilation in a wide range of community settings.
